# A “mysterious ghost kidney stone” in an 8-year-old boy with a solitary right kidney, obstructive megaureter, and ureterostomy: Answers

**DOI:** 10.1007/s00467-020-04709-x

**Published:** 2020-07-21

**Authors:** Andrzej Badeński, Omar Bjanid, Marta Badeńska, Bartosz Chmiela, Piotr Adamczyk, Grzegorz Kudela, Grzegorz Moskal, Maria Szczepańska

**Affiliations:** 1 Department of Pediatrics, Faculty of Medical Sciences in Zabrze, Medical University of Silesia, ul. 3 Maja 13/15, 41-800 Zabrze, Poland; 2 Department of Advanced Materials and Technologies, Faculty of Materials Engineering, Silesian University of Technology, ul. Krasińskiego 8, 40-019 Katowice, Poland; 3 Department of Pediatrics, Faculty of Medical Sciences in Katowice, Medical University of Silesia, ul. Medyków 16, 40-752 Katowice, Poland; 4 Department of Paediatrics Surgery and Urology, Faculty of Medical Sciences in Katowice, Medical University of Silesia, ul. Medyków 16, 40-752 Katowice, Poland

## What are common complications of high, noncontinent, urine diversions?

While a discussion of the indications and types of urinary diversion is beyond the scope of this case presentation, it should be stressed that those indications have been drastically narrowed during the last decades, and, whenever possible, primary reconstruction is preferred over temporary diversion. High diversions particularly, like Sober ureterostomy, can lead to damage to the ureter vasculature and raise the inherent problem of potentially difficult subsequent reconstruction. They should consequently be reserved for carefully selected cases [1, 2]*.*

**Fig. 1:**
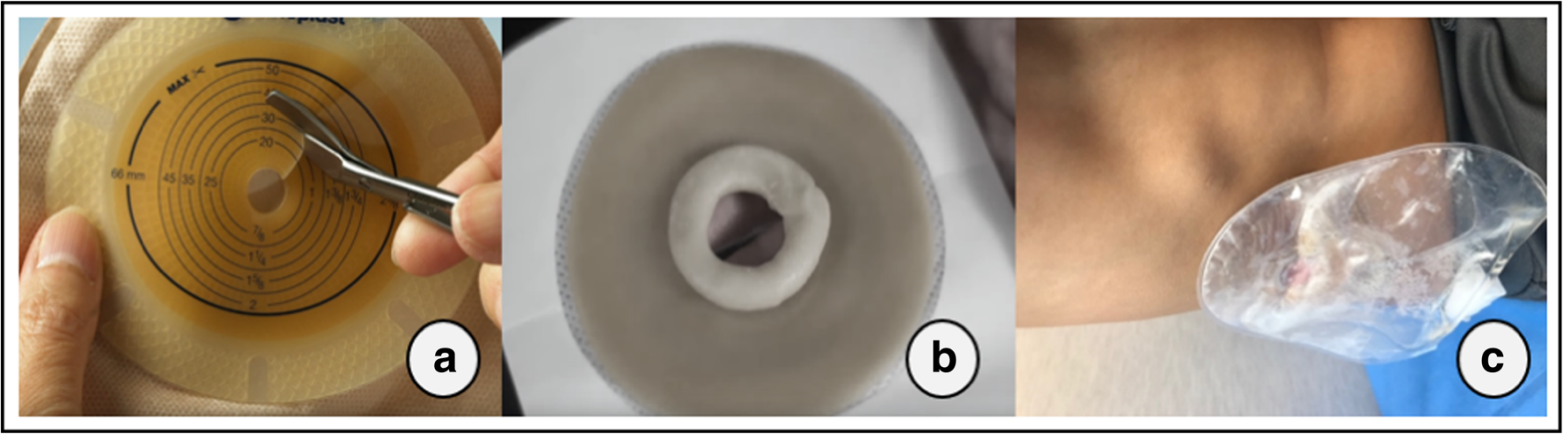
**a** Example of cut-to-fit skin barrier covered in release film. **b** Ostomy paste applied on the opening edge after removal of the film. **c** Ureterostomy pouch in the described patient

In Sober or “en Y” ureterostomy the ureter is transected, the proximal end is then anchored to the muscle, sheath, and the skin, while the distal end is connected with the lowest part of the renal pelvis to maintain the patency of the natural urinary tract and possibly preserve bladder function [3]. Frequent complications of those procedures are stomal stenosis, urinary tract infections and, commonly in noncontinent diversions, peristomal dermatitis [4]. The skin in the peristomal area constitutes a chronically occluded milieu subjected to mechanical forces and the irritating contact with urine. Skin lesions are therefore very common and include a number of dermatoses, like irritant (urine) contact dermatitis, mechanical dermatitis, chronic papillomatous dermatitis, seborrheic dermatitis, allergic contact dermatitis, as well as their infectious (bacterial and fungal) complications [5]. The risk of skin damage is minimized by proper ostomy care and collecting pouch replacement in accordance with the intended wear time or in case of urine leakage. There are different pouching systems, but basically, all ostomy appliances consist of a collecting pouch with a tap for urine outflow, and an adhesive disc-shaped part, fixed to the skin and referred to as the skin barrier or wafer, with an opening fitted to the stoma. Depending on the device, this aperture can have a fixed precut diameter, or may require the measurement of the ureterostomy “spout” diameter and the cutting of an adequate size opening (Fig. 1a). To improve the pouch’s adherence to the skin, stoma care products like sealing pastes (Fig. 1b), rings, or strips may be used on the wafer around the opening edge. Given that the skin/pouch interface is a key factor in preventing stoma effluent leakage, skin complications and overall patient’s comfort, significant effort has also been dedicated to improve appliance adhesives technology. While first generation adhesives consisted of simple zinc oxide, modern ones are of compound, sophisticated design. They usually combine hydrophobic polymers on one hand, like styrene-isoprene-styrene, polyisobutylene, or butyl rubber, which adhere to the skin and determine stickiness, adhesion and ease of removal, and on the other hand, organic hydrophilic polymers like Karaya Gum, Guar Gum, or carboxymethylcellulose, which absorb moisture, lessen skin maceration, and control erosion resistance.

## What is the final diagnosis?

Endoscopic revision of the right ureter and renal pelvis through the stoma was performed. A sticky, plastic substance, with a chewing gum like consistency as described by the urologist, was found in the right renal pelvis. Its complete removal was impossible due to its size and plasticity. The extracted material presented as drop like translucent sticky structures with a smooth surface. Those macroscopic characteristics were reminiscent of the adhesive covered part of the pouch, which prompted further investigations in collaboration with the Department of Advanced Materials and Technologies, Faculty of Materials Engineering, Silesian University of Technology, Gliwice, Poland. Fragments of the suspected foreign body were analyzed with a Hitachi 3400 N scanning electron microscope (SEM) and a Thermo Noran System Six energy dispersive spectrometry (EDS) device, to evaluate its microstructure and chemical composition.

**Fig. 2:**
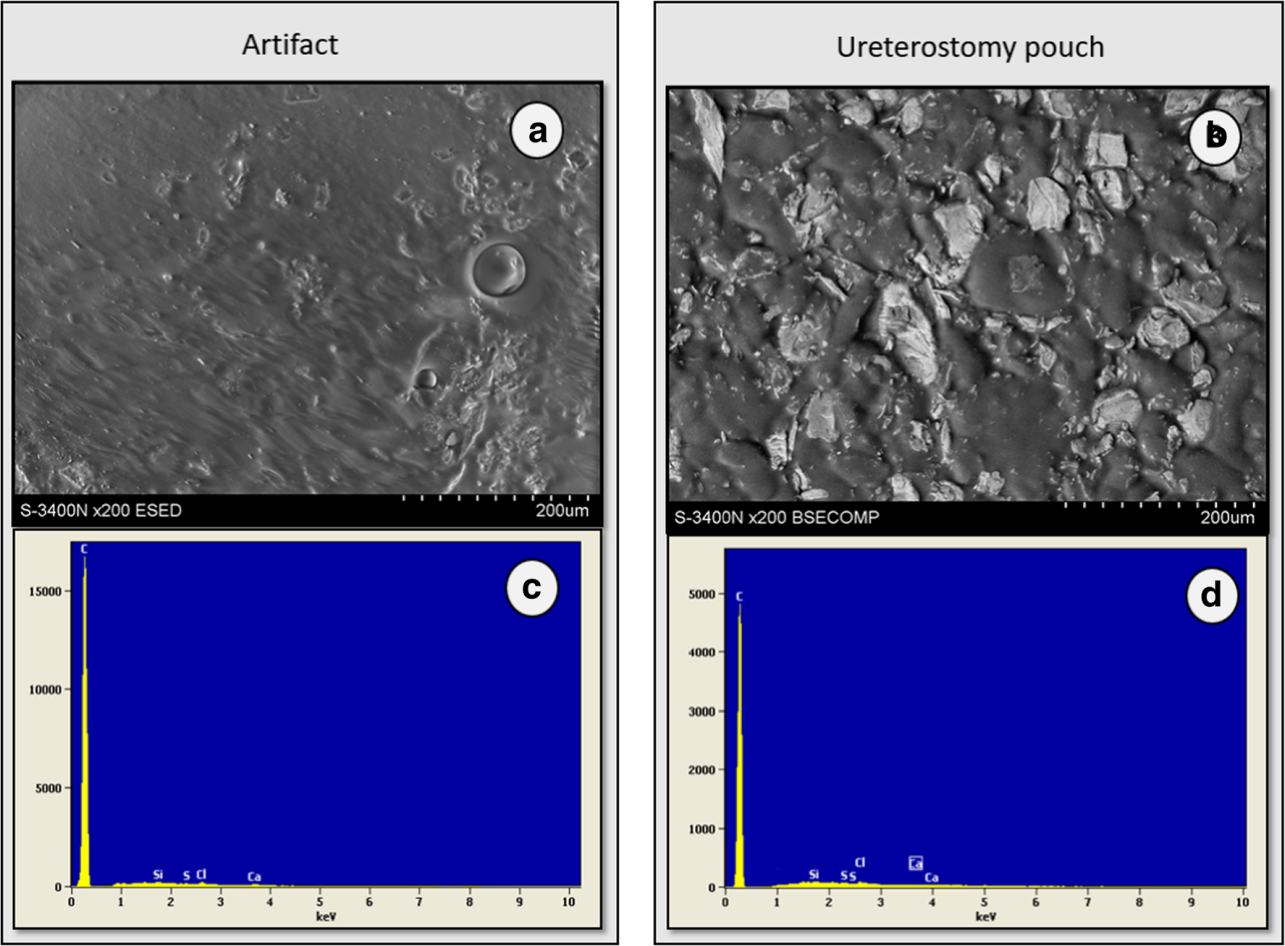
**a** Artifact surface under microscope. **b** Surface of the urostomy pouch’s adhesive skin barrier under microscope. **c** EDS spectrum of the artifact. **d** EDS spectrum of the urostomy pouch’s adhesive skin barrier

The size of the specimen and EDS method limitations allowed only for qualitative analysis of its chemical composition, which consisted of carbon, chlorine, calcium, silicon, and sulfur. The organic and probably polymeric nature of the specimen could also be assumed, which is consistent with the nature of the adhesives used in pouching devices as described above. Moreover, beside ultrastructural similarities (Fig. 2a, b), the EDS spectrum of the specimen and the pouche’s adhesive part, were practically identical (Fig. 2c, d).

Those findings strongly corroborated the hypothesis that the artifact was likely the effect of back flushing of adhesives from the skin/pouch interface to the urinary tract, and its collection in the renal pelvis and, to a lesser degree, the urinary bladder. This complication was, to our best knowledge, very rarely described and several factors probably contributed to its genesis [6].

Firstly, the ureterostomy was performed in the first year of the boy’s life, and was kept for about 8 years, when the foreign body was detected for the first time. As mentioned before, temporary cutaneous ureterostomies are rarely performed nowadays, considering that their major drawback is that subsequent urinary reconstruction can be more difficult than primary reconstructive surgery [7]. The urologist’s understandable reluctance to perform ureteral reconstruction in the presented case of a solitary kidney, progressive chronic kidney disease and doubts as to the kidney viability, led to a situation where a temporary urine diversion became pretty much permanent. In the initial clinical setting of an obstructive megaureter, and urgent indication for a urine diversion while a ureter reimplant surgery was impossible to perform, a percutaneous nephrostomy tube would arguably have been a temporary procedure less traumatic for the ureter and renal pelvis. A cutaneous ureterostomy might have been indicated in the case of posterior urethral valve and a hostile bladder, which was not the case in this patient.

Secondly, reassessment of the daily ostomy care revealed that during the appliance changing, too small an opening was cut off in the skin barrier by the child’s caregivers. That could lead to a reduction of the distance between the edges of the adhesive and sealing paste covered skin barrier, and the stoma lumen. While an inadequately large pouch opening exposes the skin to the irritating contact with urine, a too small one may promote adhesives washing out by the urine and their deposition in the pouch.

Finally, the pouch, which was daily drained and sealed before sleep, was too small in regard to the amount of urine produced during the night. Its overfilling probably facilitated the backflow of urine containing molecules of the sealing paste and adhesives to the renal pelvis, and subsequently led to the formation of the foreign body. In that respect, it is recommended to drain the pouch when it is one-third, or half filled. Alternatively, night drainage device systems are available; they are connected to the bottom pouch valve and drain urine excess, allowing undisturbed sleep while preventing pouch overfilling. It is also worth noting that many ureterostomy pouches are fitted with antireflux mechanisms, which obviously minimize the risk of the discussed complication.

## Conclusions

1. Backward urine flow from the urostomy pouch to the renal pelvis can be a source of extrinsic particles which can accumulate in the urinary tract.
2. The shadowing and twinkling artifact observed in ultrasound, usually considered as specific to kidney stones, may also be observed in some atypical circumstances, such as those described in this case [8, 9].

## Data Availability

Not applicable.
